# Nanoscale optical and structural characterisation of silk

**DOI:** 10.3762/bjnano.10.93

**Published:** 2019-04-23

**Authors:** Meguya Ryu, Reo Honda, Adrian Cernescu, Arturas Vailionis, Armandas Balčytis, Jitraporn Vongsvivut, Jing-Liang Li, Denver P Linklater, Elena P Ivanova, Vygantas Mizeikis, Mark J Tobin, Junko Morikawa, Saulius Juodkazis

**Affiliations:** 1 Tokyo Institute of Technology, Meguro-ku, Tokyo 152-8550, Japan; 10 Tokyo Tech World Research Hub Initiative (WRHI), School of Materials and Chemical Technology, Tokyo Institute of Technology, 2-12-1, Ookayama, Meguro-ku, Tokyo 152-8550, Japan; 11 Melbourne Center for Nanofabrication, Australian National Fabrication Facility, Clayton 3168, Melbourne, Australia; 2 Neaspec GmbH, Bunsenstrasse 5, 82152 Martinsried, Germany; 3 Stanford Nano Shared Facilities, Stanford University, Stanford, CA 94305, USA; 4 Department of Physics, Kaunas University of Technology, Studentu street 50, LT-51368 Kaunas, Lithuania; 5 Swinburne University of Technology, John st., Hawthorn, 3122 Vic, Australia; 6 Infrared Microspectroscopy Beamline, Australian Synchrotron, Clayton, Victoria 3168, Australia; 7 Institute for Frontier Materials, Deakin University, Geelong, VIC 3220, Australia; 8 School of Science, RMIT University, Melbourne, VIC 3001, Australia; 9 Research Institute of Electronics, Shizuoka University, Naka-ku, 3-5-3-1 Johoku, Hamamatsu, Shizuoka 4328561, Japan

**Keywords:** absorbance, anisotropy, retardance, silk

## Abstract

The nanoscale composition of silk defining its unique properties via a hierarchial structural anisotropy needs to be analysed at the highest spatial resolution of tens of nanometers corresponding to the size of fibrils made of β-sheets, which are the crystalline building blocks of silk. Nanoscale optical and structural properties of silk have been measured from 100 nm thick longitudinal slices of silk fibers with ca. 10 nm resolution, the highest so far. Optical sub-wavelength resolution in hyperspectral mapping of absorbance and molecular orientation were carried out for comparison at IR wavelengths of 2–10 μm using synchrotron radiation. A reliable distinction of transmission changes by only 1–2% as the anisotropy of amide bands was obtained from nanometer-thin slices of silk.

## Introduction

Recent advances in the nanofabrication of electronic devices require cutting-edge analytical technologies to provide a reliable structural characterisation of materials at the nanoscale. Such technologies are particularly important to probe molecular properties of cross sections smaller than 100 nm in all three dimensions, which is of rapidly growing interest in the field of nanotechnology. Electronic chip manufacturing is currently introducing the sub-10 nm fabrication node (a half pitch of a grating pattern) in the development of 3D fin-gates of field-effect transistors. Nanofabrication techniques are approaching single-digit-nanometer resolution using electron emission [1] and thermal probes [2–3]. Further control of surface nanotexturing, to achieve regularly patterned features with sub-100 nm resolution, is currently under development for inherent material properties, such as controllable surface wettability, anti-biofouling, anti-reflection, and biocidal/bactericidal properties [4–5]. For example, the motheye plastic films produced by roll-to-roll technology already replicate nanopillars with 100 nm separation (MOSMITE from Mitsubishi Chemicals Ltd.).

The structural and optical properties of a material are interrelated. By using a wide spectrum of electromagnetic waves from visible light to terahertz radiation, it is possible to gain insights into complex hierarchical structures of composite materials. For materials with strong structural anisotropy, defined by the molecular orientation and alignment of crystalline microvolumes, it is important to characterise structure at the highest lateral and longitudinal resolutions [6–7]. Anderson localisation of light and thermal cooling of silk at IR wavelengths was recently demonstrated to be related to the fibril substructure of silk, which was in the range of tens of nanometers [8]. This defines the range of the spatial resolution required for structural and chemical analyses that are typically carried out using X-ray and IR-based techniques at larger scales.

Real and imaginary parts of the refractive index, 
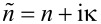
, together with the orientation dependency of the birefringence Δ*n* and dichroism Δκ, define the optical response of a material. The reflectance *R* is proportional to the real part, while the absorbance *A* corresponds to the imaginary part of 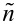. Recently, we demonstrated that the IR measurements of silk performed using three different methods, i.e., (i) a table-top Fourier-transform infrared (FTIR) transmission spectrometer, (ii) a synchrotron-based attenuated total reflection (ATR) FTIR spectrometer, and (iii) an atomic force microscopy (AFM) tip responding to the absorbed IR light (nano-IR [9]), produced comparable spectral features [10]. Whilst the first two modalities probe micrometer-sized volumes of silk, the AFM-based nano-IR technique acquires structural information at the nanoscale (i.e., the area under the AFM tip from a volume with a lateral cross section of ca. 20 nm). Differences in absorbance and spectral line shapes of the characteristic silk bands are related to the different sensitivity of *R* and *A* to the real and imaginary parts of 
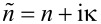
. The absorbance measured from the far-field transmission directly reflects the imaginary part of the index κ, while the absorbance obtained in the ATR-FTIR mode is affected by the real part of the index via Snell’s law [11]. As a result, comparative measurements of the absorbance by different near- and far-field techniques are essentially required to understand differences in electric-field determination of the local light and its interaction with the sample [12].

Different modalities of sample preparation for nanoscale imaging include focused ion beam milling and microtome slicing. When the thickness of samples, especially soft biomaterials, is close to 100 nm the cutting tool might cause tear- and cut-induced strain below the surface. In turn, this can cause artifacts in the determination of optical properties that are related to the mass density and its gradients. It is important to measure *n* and κ from decreasingly smaller volumes and to compare with data obtained from the bulk samples.

Here, we used a near-field scattering method to probe *n* and κ and to determine spectral differences between the reflectance and absorbance of silk fibers with ca. 10 nm resolution. Cross sections of silk fibers were prepared using an ultramicrotome. Silk was chosen due to its well-known spectral properties and its increasing applications as a biocompatible and biodegradable material [13–14]. Silk exhibits a uniaxial symmetry that can be examined from longitudinal microtome slices used in this study. Sub-wavelength resolution in hyperspectral IR mapping of absorbance and orientational properties of the absorbing bands was reliably achieved in 100 nm thick slices of silk. Such a high-resolution technique is essential in order to gain a better understanding of the fibril structure of silk [8].

## Experimental

### Silk slices

White *Bombyx mori* cocoons were purchased from the silk rearing house in Jiangsu, China, and brown *Antheraea pernyi* silkworm cocoons were collected from Liaoning Province, China. The white and brown silk fibers used in this work are fibroin fibers obtained by degumming *Bombyx mori* and *Antheraea pernyi* silk fibres, respectively. To degum the fibres, the cocoons were boiled three times in an aqueous 0.5% (w/v) Na_2_CO_3_ solution to remove the sericin coating. The degummed silk fibers were rinsed with warm ultrapure water (60 °C) thoroughly to remove the residual sericin, and then dried at room temperature.

Silk fibers were embedded in epoxy resin (Oken Ltd., Japan) and cut by using an ultramicrotome to achieve a sample thickness of ca. 100 nm. The slices were then immobilised on IR-transparent non-birefringent CaF_2_ substrates.

### X-ray characterization

**Figure 1:**
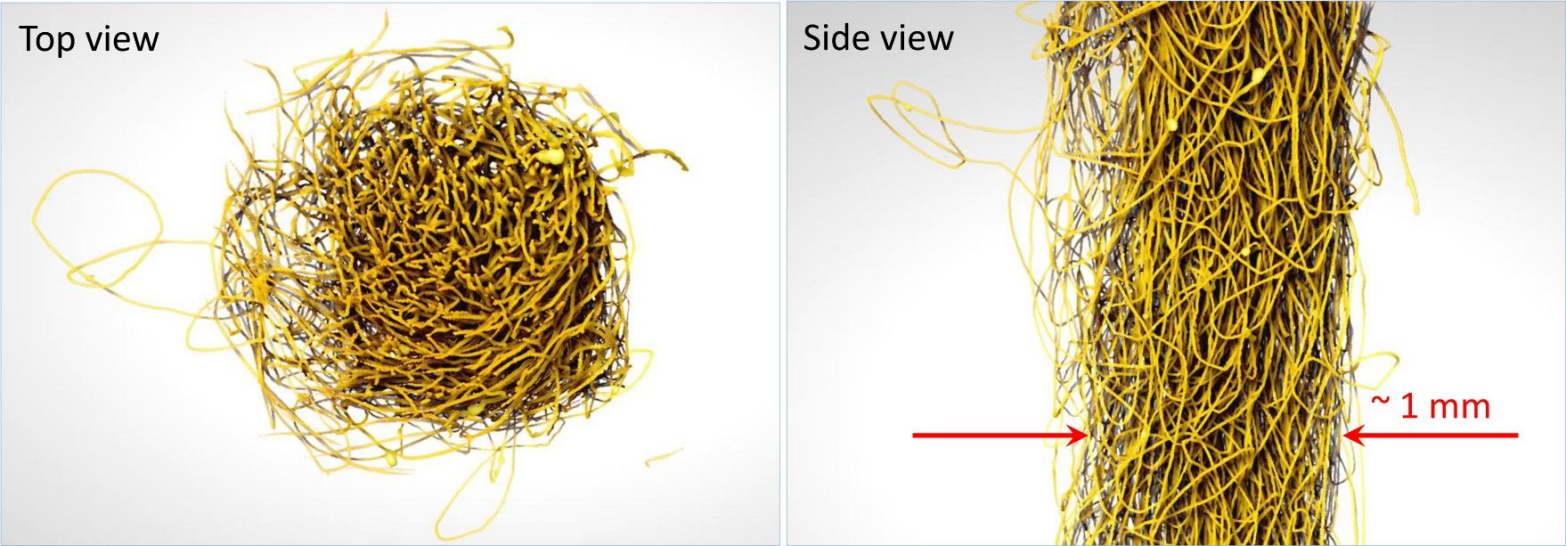
X-ray tomographic images showing 3D rendered volumes of white *Bombyx mori* silk fibers at 3.15 μm pixel resolution. The bundle of silk fibers is composed of degummed single-strand silk fibers.

3D X-ray computed microtomography (micro-CT) of white *Bombyx mori* silk fibers was performed using a ZEISS Versa 520 X-ray Microscope at the Stanford Nano Shared Facilities, Stanford University. The scan settings were as follows: source voltage - 30 kV, pixel size - 3.15 μm, number of projections - 1600, exposure time - 10 s. The micro-CT dataset was reconstructed using the ZEISS Scout-and-Scan Reconstructor software (Figure 1).

**Figure 2:**
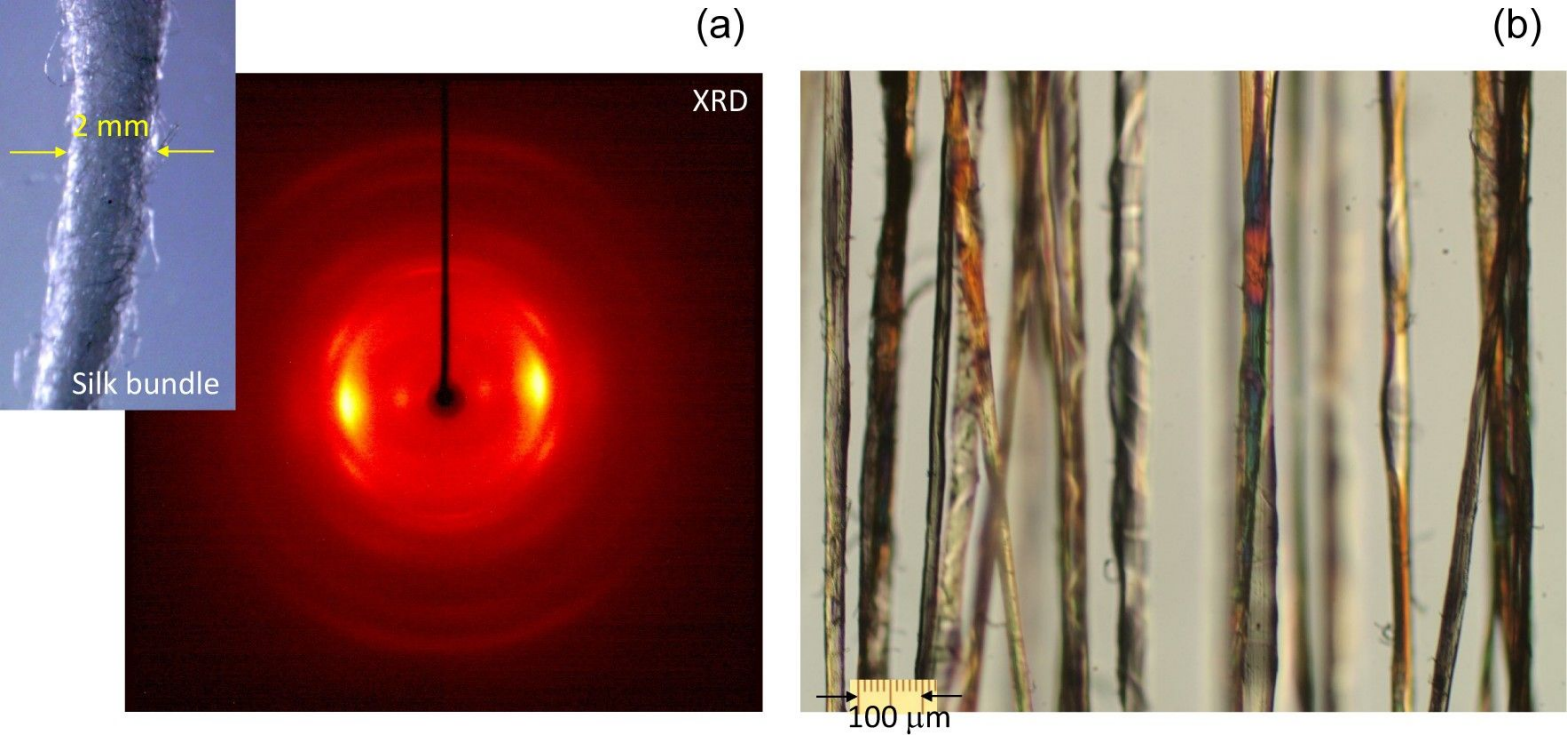
(a) Wide-angle 2D X-ray diffraction of a bundle of white *Bombyx mori* silk fibers. The inset shows an optical microscopic image of a convolved silk fiber bundle. The silk bundle was composed of degummed single-strand silk fibers. The long axis of the fibers was predominantly vertical. (b) Optical image of white silk fibers through an optically aligned polariser–analyser (high-transmission) setup under white-light illumination using a Nikon MPlan 10× DIC objective lens with numerical aperture NA = 0.25.

2D X-ray diffraction of *Bombyx mori* silk was carried out on a Bruker D8 Venture single-crystal diffractometer using a Cu Kα microfocus X-ray source with λ = 1.5418 Å (Figure 2a).

### IR spectral measurements

The sub-diffraction scattering scanning near-field optical microscope (s-SNOM, neaspec GmbH) uses a metalized atomic force microscopy (AFM) tip. The tip maps the surface relief (topography) by its basic AFM operation and, simultaneously, under external infrared illumination (broadband laser with difference frequency generation, Toptica), acts as a light-concentrating antenna such that the sample is probed with a nanofocused light field. The AFM tapping-mode operation (ca. 60 nm amplitude) modulates the near-field interaction between the tip and sample [15]. An asymmetric Michelson interferometer and a lock-in detection of the signal at higher harmonic of the tapping frequency (approximately 250 kHz) provides background-free nano-IR spectra and images with maximum resolution imposed by the AFM tip size independent of the laser wavelength [12].

The nano-FTIR spectra were recorded at a rate of ca. 100 s/spectrum with a spectral resolution of 10 cm^−1^. Removal of the instrumental response function from the nano-FTIR spectra was done by normalization of the measured spectra to a reference Si signal. Resulting nano-FTIR absorption and reflectivity spectra can be directly correlated with the standard far-field IR spectra [16–17].

Hyperspectral imaging of the absorbance was measured on the IR Microspectroscopy (IRM) Beamline at Australian Synchrotron (Victoria, Australia). The measurements were performed using a Bruker Hyperion 2000 FTIR microscope (Bruker Optik GmbH, Ettlingen, Germany) coupled to a Vertex V80v FT-IR spectrometer equipped with a liquid nitrogen-cooled narrow-band mercury cadmium telluride (MCT) detector. Holographic ZnSe wire-grid polarisers (Edmund) were used to set polarisation at the IR spectral range of λ = 750–4000 cm^−1^ (2.5–13.3 μm); the extinction of polarisers was *T*^max^/*T*^min^ ≈ 150 and the transmittance was about 50%. The far-field transmission measurements were carried out with a 36× magnification Cassegrain objective lens (NA = 0.5) at the corresponding resolution of 0.61λ/NA ≈ 4.1 μm at the 3000 cm^−1^ band (λ = 3.33 μm). The absorbance or optical density *A* = −log(*T*) spectrum is defined by the absorption coefficient α ≡ 4πκ/λ = 2ωκ/*c* [cm^−1^] for the transmitted light intensity *I*_T_ = *I*_0_*e*^−α^*^d^* = *I*_0_ × 10^−OD^; where *d* is the thickness of sample, the transmittance *T* = *I*_T_/*I*_0_, OD is the optical density, ω is the cyclic frequency of light, and *c* is the speed of light. The reflectance for the normal incidence from air is defined as *R* = [(*n*− 1)^2^ + κ^2^]/[(*n* + 1)^2^ + κ^2^].

## Results and Discussion

X-ray diffraction is the method of choice to reveal the internal structure of complex materials and to detect crystalline regions. Figure 1 and Figure 2a show 3D reconstructions of the *Bombyx mori* silk fibers bundled together and their X-ray diffraction (XRD) pattern, respectively. The period *d* corresponds to the most pronounced peaks at the diffraction angle 2θ, given by Bragg’s law *d* = λ/(2sinθ). The size *L* of the nanocrystalline phase can be estimated from the Scherrer equation *L* = *K*λ/(*B*(2θ)cosθ; where *K* = 0.89 for spherical crystals and *B*(2θ) is the full width at half maximum of the peak. The wide-angle XRD pattern (Figure 2a) is identical to that reported earlier [18]. The most pronounced peak corresponds to the separation between the equatorial (200) planes *d*_(200)_ = 4.69 nm and crystal cross section of *L* ≈ 2.15 nm, while for the meridional (002) planes *d*_(002)_ = 3.46 nm and crystal size of *L* ≈ 10.76 nm [18]. These are the dimensions of the β-sheets, which are crystalline segments in the silk fiber. SNOM measurements are well suited to measure *n* and κ from areas of comparable dimensions.

**Figure 3:**
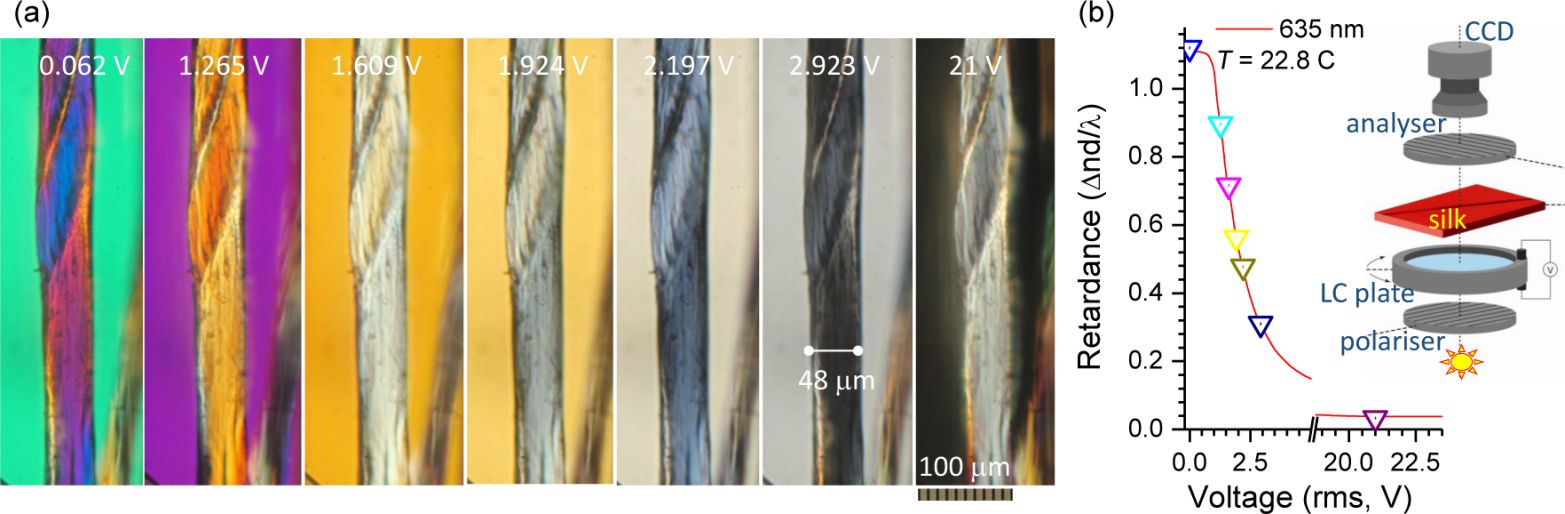
(a) A series of optical images taken at different voltages of a liquid crystal (LC) retarder (schematically shown in the inset of (b)) and a Nikon Optiphot-pol microscope with LMPlanFL 20× objective lens, NA = 0.4. (b) Calibration curve of retardance as a function of voltage collected at 635 nm wavelength and 22.8 °C.

Silk is a strongly birefringent material, as revealed by cross-polarised optical imaging (Figure 3). The images were taken following adjustments of the voltage of a liquid crystal (LC) retarder, which was inserted with its slow-axis perpendicular to the orientation of the silk fiber (see inset in Figure 3b). Using such a geometry, it is possible to compensate for the birefringence of the silk fibers, Δ*n* ≡ *n*_e_ − *n*_o_ *>* 0, with a phase delay imparted by the LC retarder. When the phase delay through the LC retarder is equal to the absolute value, but has an opposite sign through the silk fiber, the darkest (black) region is formed in the image at ca. 2.9 V (Figure 3a). For the thickness of fiber *d* = 48 μm and measured retardance, the birefringence Δ*n* ≈ 4 × 10^−3^. This is an estimate of the order of magnitude since the calibration of the LC retarder is carried out at a single wavelength, while the imaging is done under white-light illumination. The birefringence originates from the alignment of the structures, which is determined by the fiber orientation down to molecular bonds and spans hierarchically over a wide range of wavelengths due to secondary ordering [19]. Previously, longitudinal ca. 1 μm thick silk slices were measured in transmission mode using synchrotron IR radiation to characterise the molecular alignment of the typical amide bands [20], including amide II at 1512 cm^−1^ (C–N), amide I (β-sheets) at 1628 cm^−1^ (C=O), and amide A at 3290 cm^−1^ (N–H). A perpendicular orientation between C=O and C-N bonding was revealed at a high accuracy when longitudinal silk slices were prepared [20]. Longitudinal slices facilitated more precise measurements of the molecular alignment since there were no averaging artifacts due to the curvature of silk fiber and different thickness across the fiber slice [21].

**Figure 4:**
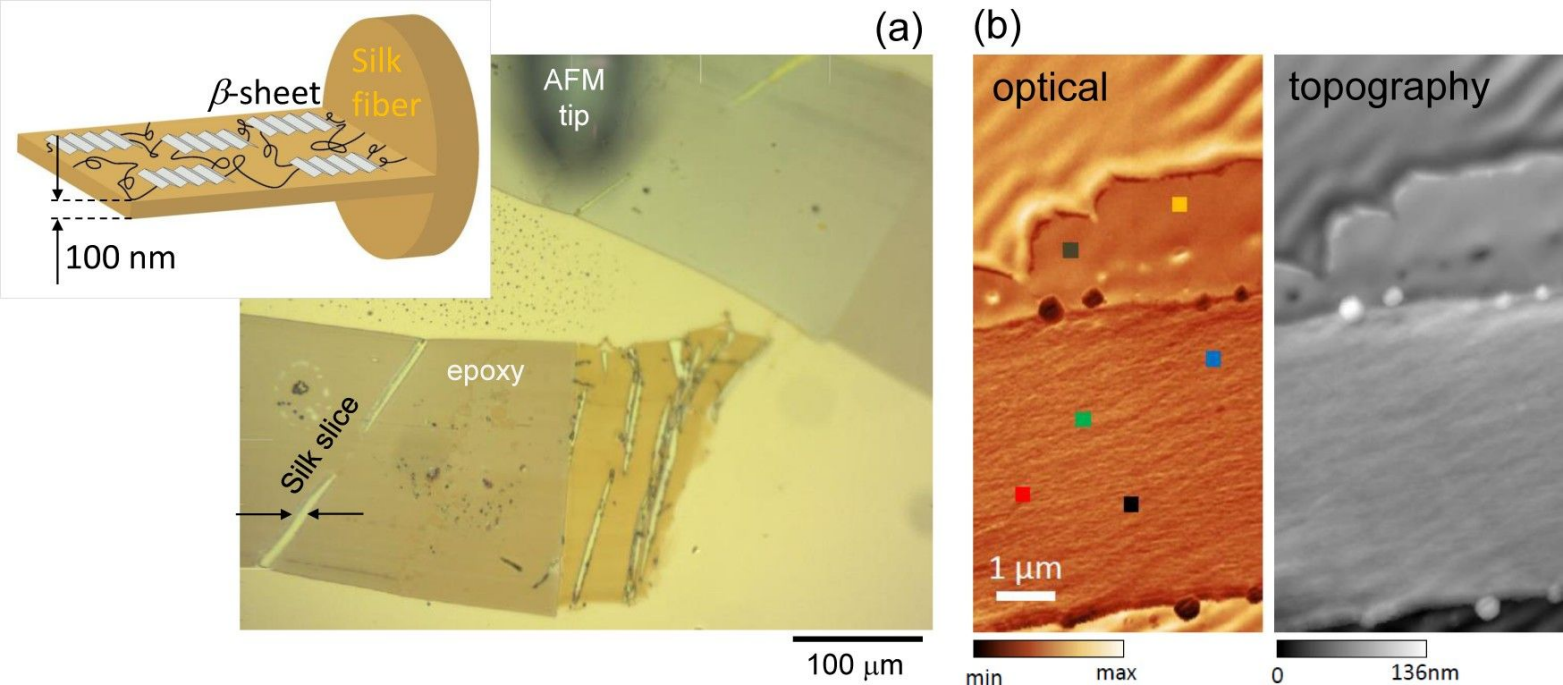
(a) Far-field optical image of longitudinal slices of white silk embedded in an epoxy sheet. The inset shows schematics of a lateral silk slice composed of β-sheets interconnected with α-coils and amorphous segments. (b) Optical and topographic images of the silk slice shown in (a) measured with scattering near-field microscopy (SNOM; neaspec). Markers in optical image indicate locations where spectra were acquired.

**Figure 5:**
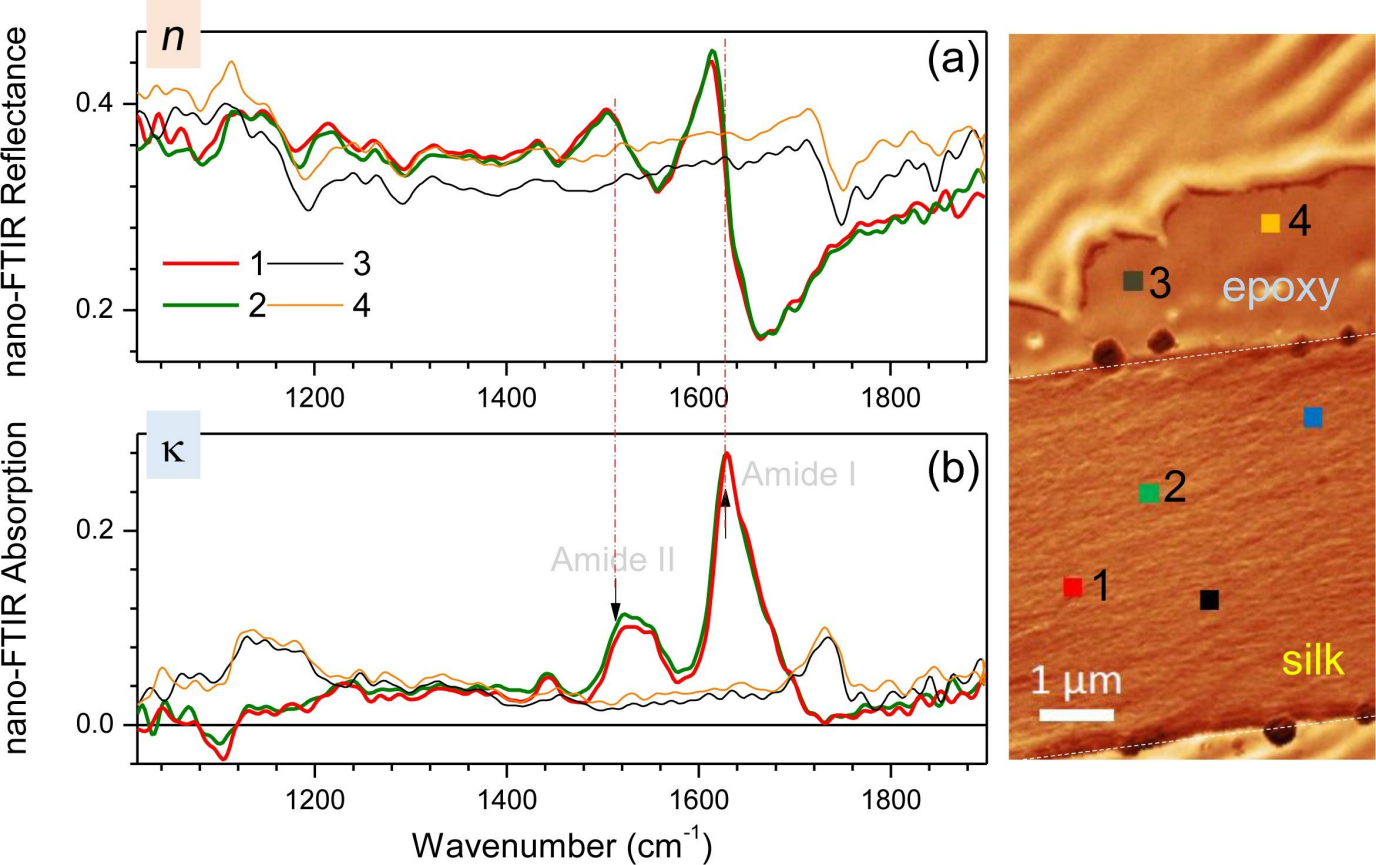
Scattering near-field optical microscopy (SNOM) measurements of the nano-FTIR reflectance (a) and absorption (b) spectra from selected points on silk and epoxy (shown in the right inset).

Scattering SNOM was used to measure reflectance and absorbance spectra from nanoscale areas of a single silk slice. Lateral slices of 0.1 μm were prepared on a gold mirror (Figure 4a). Optical and topographic images were obtained that confirmed the thickness of the silk slices to be ca. 100 nm (Figure 4b). Spectra of nano-FTIR reflectance and absorption from selected points were also measured (Figure 5) with a high reproducibility, showing a clear distinction between the silk and the epoxy host matrix. The nano-FTIR absorption is proportional to the imaginary part of the scattering coefficient 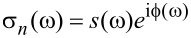, which relates the scattered field of the light *E*_s_(ω), and the incident field *E*_i_(ω) through the equation *E_s_* = σ*_n_E*_i_; where *s*(ω) and 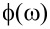 are the amplitude and phase of the back-scattered spectra [12]. The reflectivity information is given by the real part of the scattering coefficient [12]. Using an asymmetric Michelson interferometer, the full complex function of the scattered optical signal could be recorded, therefore enabling the simultaneous measurement of both nano-FTIR absorption and reflectivity spectra, shown in Figure 5.

The amide-I and amide-II bands were well reproduced in the absorption spectra collected from four different single points. However, only spectra from two measurement points are displayed in Figure 5 for a better clarity of presentation. Nanoscale resolution is readily achievable for SNOM measurements and is defined by the AFM tip, which has a diameter of ca. 10 nm. Around the center of the absorption peak, regions of normal dispersion with a higher refractive index at a higher photon energy (proportional to the wavenumber) was observed. Spectral positioning of the absorption peak and dispersion line shapes corresponded to the expected Lorentzian behavior of a damped oscillator.

**Figure 6:**
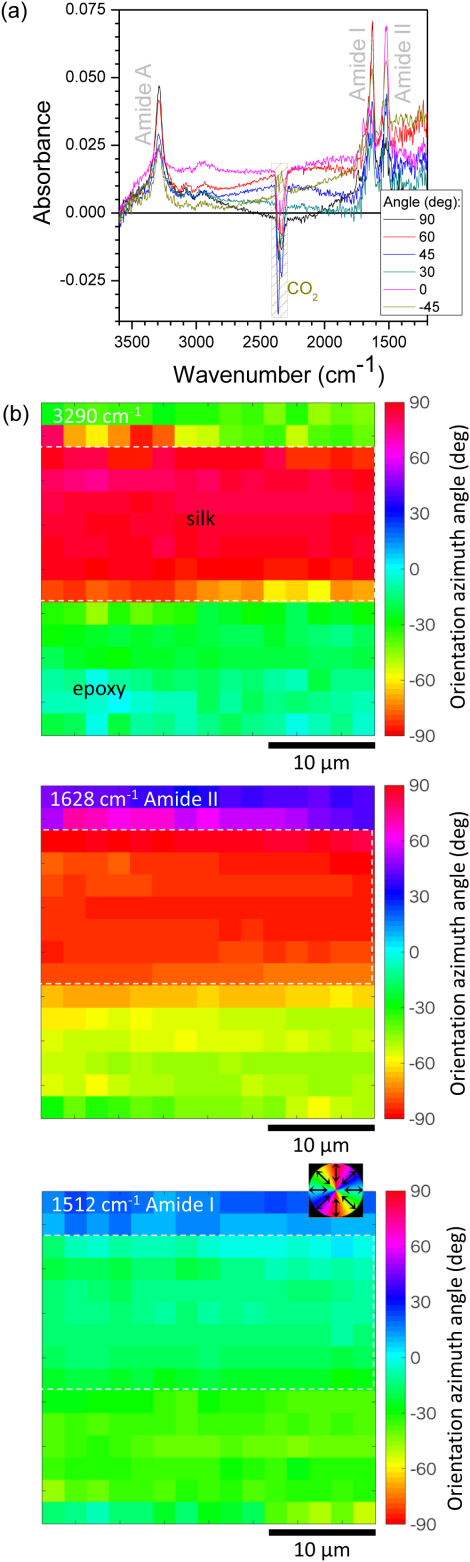
(a) Single-point absorbance spectra of thin silk samples on BaF_2_ collected at different angles θ between the linear polarisation and the fiber axis, using 2 μm pixel pitch, 15 × 15 pixel points, 4.17 μm spatial resolution, and 4 cm^−1^ spectral resolution. (b) Orientation color maps indicating that amide A (N–H) is oriented perpendicular to amide I (C=O) and amide II (C-N).

Next, direct absorbance and orientation mapping [22] through a 100 nm thick silk slice was demonstrated using synchrotron IR radiation (Figure 6). By measuring the absorbance at several azimuth angles, θ, it was possible to determine the molecular alignment within the fibril structure. Here, we demonstrate the use of the technique on the thinnest silk section of 100 nm. The well-aligned amide bands were measured in transmission mode at wavelengths that are much longer than the thickness of the silk slice (*d* = 100 nm). A wavenumber of 1500 cm^−1^ corresponds to a wavelength of 6.67 μm. The pitch between measurement points was 2 μm and was approximately two-times smaller than the focal spot (4.1 μm). This caused an uncertainty in orientation azimuth at the boundary of the silk fiber and the surrounding epoxy matrix. However, the central part of the fiber shows a well-defined orientation, while the epoxy region has a random orientation. The absorbance from silk, which makes only *d*/λ ≈ 1.5% of the probing wavelength, was reliably measured in transmission. The retardance of silk, *d* = 100 nm, has a birefringence of Δ*n* = 4 × 10^−3^ at the non-absorbing vis–IR wavelengths. For example, the band at 3600 cm^−1^ (λ = 2.78 μm) resulted in Δ*T* = sin^2^(πΔ*nd*/λ) = 2 × 10^−5^%, which was beyond the precision of measurements. Alternatively, the real part of the refractive index can be determined from the known values of reflectance *R* and extinction κ following the equation *n* = [(1 + *R*)/(1 − *R*)] + [4*R*/(1 − *R*)^2^ − κ^2^]*^1/2^*. However, *R* was not measured in this experiment.

Anisotropy in absorption is defined by the dichroism


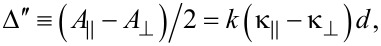


where *k* = 2π/λ is the wave vector. It defines the losses in transmission *T*, at the maximum and minimum orientations of linear polarisation 
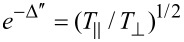
. The dichroism was estimated for the amide bands. For the amide-A band, Δ” ≈ 0.014 suggests only a minute transmission change 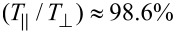 for the two perpendicular polarisations. Similarly, the results obtained for the amide-I band (Δ” ≈ 0.027 and 97.4%) and the amide-II band (Δ” ≈ 0.019 and 98.1%) also indicated that very small changes of absorbance of light passed through the thin 100 nm lateral slices of the silk fiber occurred. This shows that an anisotropy of absorbance can be measured from nanoscale materials of sub-wavelength thickness. There were no apparent spectral differences among the measurements at different orientations of 100 nm thick silk slices. The far-field (Figure 6) and near-field (Figure 5) absorbance spectra are comparable and are matching earlier results measured from thicker samples [10]. This study shows that the SNOM measurements reach the resolution required to measure the structural composition of silk fibres corresponding to the crystalline segments observed in XRD and the measurements can be carried out with nanometer-thin slices of silk.

## Conclusion

Spectral characterisation, lateral mapping and transmission with deep sub-wavelength resolution in the spectral window of IR molecular fingerprints were demonstrated using 100 nm thin lateral slices of silk. Absorbance and reflectance spectra of silk with the resolution of the SNOM tip of ca. 10 nm were obtained. Absorbance from nanometer-thin silk slices with thickness only 1.5% of the wavelength were measured when the beam diameter was comparable to the IR wavelength. Hyperspectral mapping across the silk fiber slice was obtained with high accuracy and reproducibility. An orientational map of the amide bands was revealed and was consistent with data collected from bulk samples. It shows that preparation of thin microtome slices of soft biomaterials is not altering their structure and opens the possibility to read optical properties from nanovolumes. In the case of optical measurements, optical averaging over thicker inhomogeneous volumes of samples can be avoided using nanoslices and this provides more reliable direct measurement of optical properties. The study demonstrated the characterisation of silk fibers with nanoscale resolution in all three dimensions.
